# Arterial pressure‐guided management following cardiopulmonary resuscitation

**DOI:** 10.1002/clc.23501

**Published:** 2020-11-12

**Authors:** Kosuke Miki, Teruhiko Imamura

**Affiliations:** ^1^ University of Toyama Toyama Japan; ^2^ Second Department of Internal Medicine University of Toyama Toyama Japan

To the Editor,

Neurological prognosis is one of the great concerns for the survivors of cardiopulmonary resuscitation. Ai and colleagues demonstrated that the higher mean arterial pressure (MAP), as far as below 100 mmHg, was associated with better neurological function among the survivors of cardiopulmonary resuscitation.^1^ Several concerns have been raised.

The timing of MAP measurement might be unclear. Their findings would further improve when the authors identify the most appropriate timing of MAP measurement: for example, on admission to intensive care unit (ICU), on discharge from ICU, or the average of whole ICU stay. Association between MAP and imaging analyses at serial time points might also more clarify the impact of MAP on neurological function.

The authors found the association between MAP and neurological function,^1^ but the causality between them remains unknown: it is unclear whether MAP is a risk factor of neurological impairment or a therapeutic target. Aggressive intervention to increase MAP might prevent future neurological impairment. Given that the epinephrine total dose was a risk factor of neurological impairment,^1^ methodology to increase MAP should also require further discussion.

The authors stated that the cessation of blood flow during a cardiac arrest results in the formation of microvascular thrombosis that impairs the cerebral vascular autonomic regulation system.^1^ Aggressive anticoagulation therapy during cardiopulmonary resuscitation might prevent such comorbidity.

## CONFLICT OF INTEREST

Teruhiko Imamura receives grant support from JSPS KAKENHI: JP20K17143.
